# micROS: a morphable, intelligent and collective robot operating system

**DOI:** 10.1186/s40638-016-0054-y

**Published:** 2016-11-25

**Authors:** Xuejun Yang, Huadong Dai, Xiaodong Yi, Yanzhen Wang, Shaowu Yang, Bo Zhang, Zhiyuan Wang, Yun Zhou, Xuefeng Peng

**Affiliations:** State Key Laboratory of High Performance Computing (HPCL), Computer School, National University of Defense Technology, 137 Yanwachi Street, Changsha, China

**Keywords:** micROS, Robot operating system, OODA, Collective intelligence

## Abstract

Robots are developing in much the same way that personal computers did 40 years ago, and robot operating system is the critical basis. Current robot software is mainly designed for individual robots. We present in this paper the design of micROS, a morphable, intelligent and collective robot operating system for future collective and collaborative robots. We first present the architecture of micROS, including the distributed architecture for collective robot system as a whole and the layered architecture for every single node. We then present the design of autonomous behavior management based on the observe–orient–decide–act cognitive behavior model and the design of collective intelligence including collective perception, collective cognition, collective game and collective dynamics. We also give the design of morphable resource management, which first categorizes robot resources into physical, information, cognitive and social domains, and then achieve morphability based on self-adaptive software technology. We finally deploy micROS on NuBot football robots and achieve significant improvement in real-time performance.

## Background

 The third industrial revolution is under its way [1]. Robots, as one of the most remarkable novel products in this revolution, will repeat the history of the rising of personal computers and enter every home in a near future [2]. The most important system software for robots, robot operating system, will be the key driving force for this trend. It is able to effectively solve the major problems of low modularity and standardization level faced by current robotic technology, in order to simplify software design, improve software quality, promote the integration of new technologies and reduce production costs.

Before the concept of robot operating system was introduced, system software with the same functionalities was referred to as robotics middleware, robot software framework, or robotics development environment. They gained more and more attentions and research efforts since late 1990s, with typical initiatives gradually emerging, such as Miro [3], Orca [4], RT-Middleware [5], Player/Stage [6], MARIE [7], RSCA [8] and Orocos [9]. Microsoft also released Robotics Developer Studio [10] in 2006. In 2007, the release of Robot Operating System (ROS) version 1.0 [11] introduced the concept of operating system into robotics for the first time. In recent years, this concept was gradually accepted by both academia and industry. An increasing number of experimental and commercial robot systems base their research and development entirely or partially upon robot operating systems, even including examples like the Aircrew Labor In-cockpit Automation System (ALIAS) [12] and Robonaut 2 [13], which requires very high real-time performance and reliability. However, most of the aforementioned robot operating systems mainly focused on development of applications on individual robotic platform, despite the fact that the vast majority of them support networking. It still remains an open issue for existing robot operating systems how to manage the heterogeneous resources and complex behaviors of collective robot systems to achieve collective intelligence.

In order to solve the above-mentioned problems and better adapt to the emerging of co-robot (i.e., cooperative robots) that would profoundly interact with the human society, this paper proposes the idea and design of a morphable, intelligent and collective robot operating system, micROS. The main contribution of this paper is fourfold.

- The design choice of micROS is based on autonomous behavior and collective intelligence, with management of autonomous and collective robots as its major target;
- The micROS architecture, in terms of both the distributed collective architecture for collective robots and the layered architecture for individual robots, is proposed;
- We combine the observe–orient–decide–act (OODA) cognitive behavior model and collective intelligence in the high-level architecture design and tackle the four major challenges, i.e., autonomous observation and collective perception, autonomous orientation and collective cognition, autonomous decision and collective game, autonomous action and collective dynamics;
- The morphable and adaptive mechanism of micROS is designed based on adaptive software techniques.

The remaining part of this paper is structured as follows. “micROS Architecture” section introduces the architecture of micROS. “Autonomous behavior and collective intelligence” section presents the mechanisms to implement autonomous behavior and collective intelligence. “Morphable and adaptive mechanism” section describes the morphable and adaptive design of micROS. Practical application of micROS in a RoboCup system (soccer robots), as well as the corresponding experimental results, is presented in “Application and experiments” section. Finally, “Conclusion and future work” section concludes the paper and discusses about potential future work.

## Methods

### micROS architecture

Derived from the organization structures of collective robots, we designed for micROS the overall distributed architecture and the layered structure for individual node.

#### Organization structures for collective robots

**Fig. 1:**
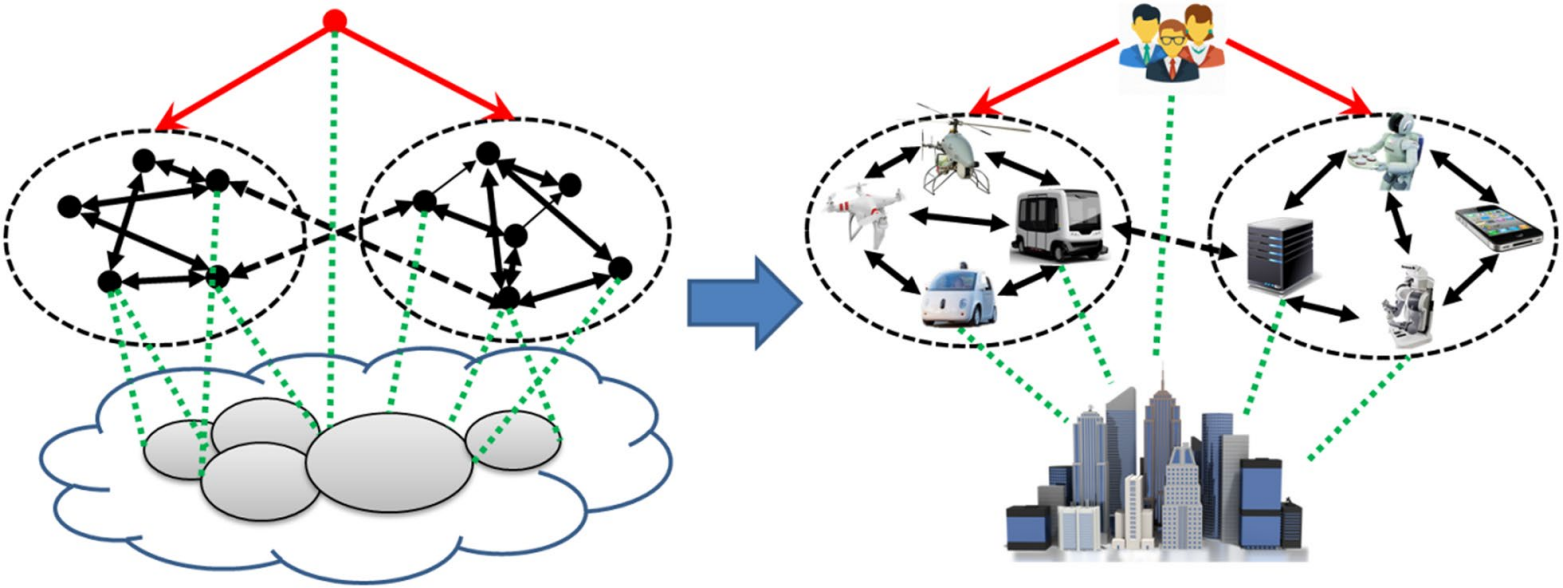
Illustrative examples for collective organization structures (*left*) and co-robot collections in a city scenario (*right*), respectively

Collective behaviors are deeply affected by organization structures, which are made of roles, relations and privileges. There are various organization structures for collective robots under different tasks and environments, such as hierarchies, holarchies, coalitions, teams, congregations, federations, markets, matrices and societies [14]. A robot, a computer or even a human can serve as a node in the organization structures. The left part of Fig. 1 illustrates an example of organization structures. It consists of an environment, several nodes in two domains and a node dominating others. All nodes together form a collection by collaborating with each other and interacting with the environment. The right part of Fig. 1 shows an example of co-robot collections with two domains serving humans collaboratively in a city scenario. In the outdoor domain, unmanned aerial vehicles and unmanned ground vehicles collaborate to percept, plan and share data to improve mobility. In the indoor domain, robots, computers and smart terminals collaborate to provide a better service.

#### The distributed architecture of micROS

**Fig. 2:**
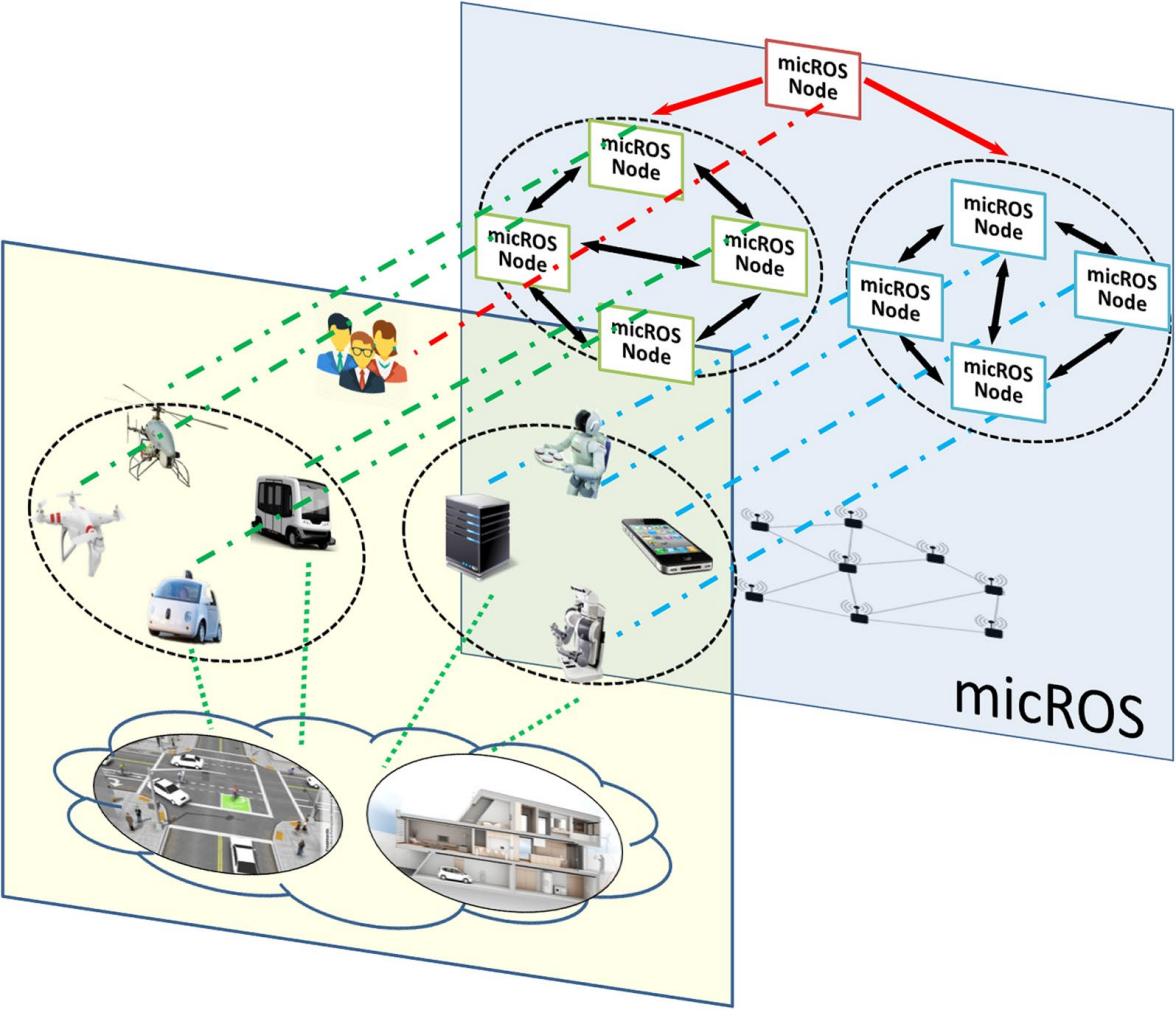
Distributed architecture of micROS for the city scenario example

Inspired by organization structures, micROS is designed to be a distributed architecture which consists of lots of individuals (nodes) interconnected. The nodes could be robots, computers or humans. micROS is installed on every node to form a distributed system, which is responsible for management of resources and behaviors, coordination of node–node and node–environment interaction and self-organization in dynamic and open environment. Figure 2 shows how to map the city scenario example to micROS.

The distributed architecture of micROS implements “inter-connecting, inter-communicating, interoperability, inter-understanding and inter-obedience.” Interconnecting and intercommunicating are implemented through distributed networking. Interoperability is supported by standardization, modularity and platformization. Inter-understanding includes three aspects: human–robot, robot–robot and robot–environment. Inter-obedience is expressed by rules in physical, information and social domains.

Networking is the basis for constructing the distributed architecture. Wireless network is the mainly used one for mobile robots. micROS will support scalable self-organizing networks based on wireless communication and provide mechanisms for robust interoperability.

Real-time guarantee is a distinguished feature of micROS. micROS will implement three levels of real-time guarantee, i.e., node-level real time, message-level real time and task-level real time. Node-level real-time guarantee is achieved by high-resolution timer, interrupt/event priorities, resource scheduling, non-blocking communication, etc. Message-level real-time guarantee is achieved based on the network protocols, such as RT-NET, which provide real-time support. Task-level real-time guarantee supports real-time constraint exchange among interconnected nodes and real-time behavior for the entire system.

#### The layered structure for micROS Nodes

**Fig. 3:**
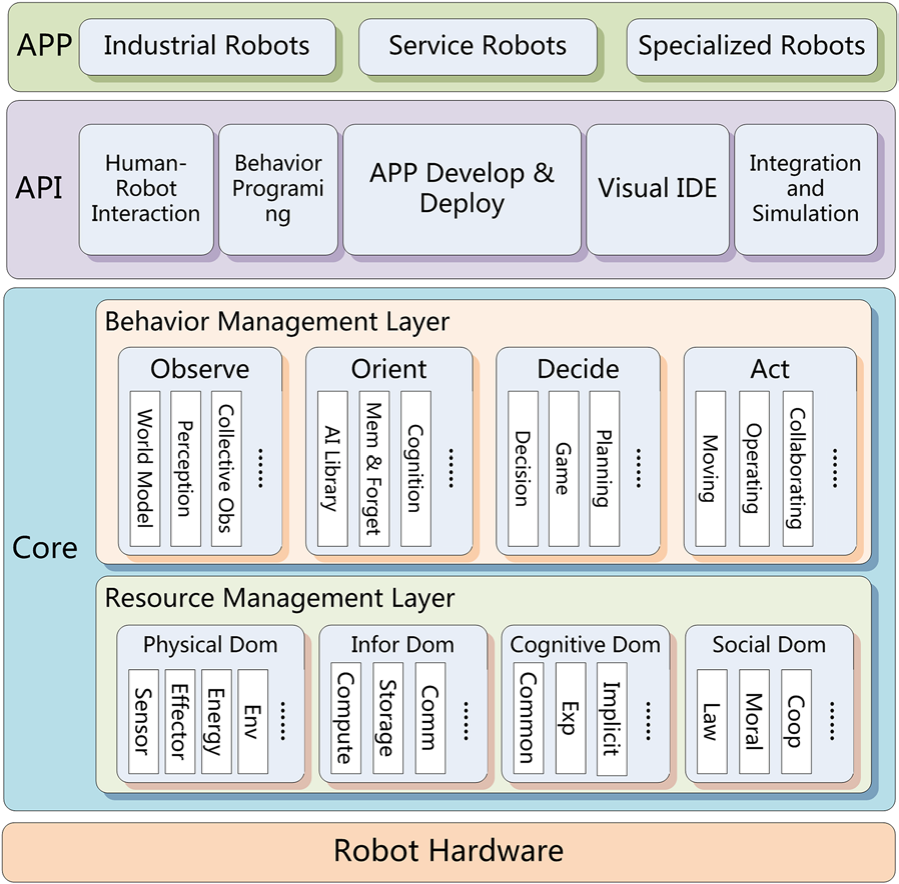
Layered structure of micROS nodes

micROS is installed on each node of the collective robots and exhibits the layered structure for each node, as shown in Fig. 3, which consists of the core layer and the API layer. The API layer is responsible for interaction and programming interface. The micROS core is divided into resource management layer and behavior management layer. The former aims at resource management in physical, information, cognitive and social domains. According to the OODA cognitive behavioral model (“Collective behavior of robots” section), the behavior management layer is composed of observation, orientation, decision and action modules.

### Autonomous behavior and collective intelligence

#### Collective behavior of robots

The OODA loop is a famous cognitive behavioral model proposed by John Boyd [15]. It was initially used to model strongly confrontational behaviors and then extended to model more general behaviors such as those in commercial and social domains.

**Fig. 4:**
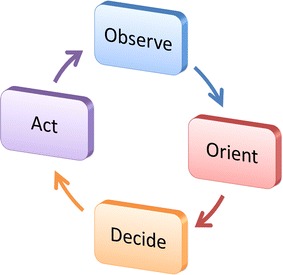
OODA loop

The OODA loop is illustrated in Fig. 4, which consists of the observe, orient, decide and act elements. It fully takes into account the nonlinearity, uncertainty, emergence, self-organization and creation characteristics of dynamic environments and complex systems. So it is capable of describing high-level and complex collective behaviors [15].

In order to implement behavior management for collective robots, micROS embodies collective intelligence into the OODA loop in four aspects: (1) autonomous observation and collective perception, (2) autonomous orientation and collective cognition, (3) autonomous decision and collective game and (4) autonomous action and collective dynamics. They will be explained in detail in the following sections.

#### Autonomous observation and collective perception

micROS manages different perception roles and their corresponding perception capabilities of each collective robot to implement autonomous observation. Moreover, micROS achieves collective perception by fusing information from all individuals of the collective robots according to the given tasks and scenarios.

In order to fuse perception information according to the different robot roles, micROS first defines the roles for each individual robot. micROS allows different ways for defining roles. For example, as defined in Webster’s dictionary, a role can be a function or a part performed function in a particular operation or process. We can also consider the role as a function that one or more robots perform during the execution of a cooperative task as in [16]. micROS assigns roles to collective robots according to the actual perception capabilities of their sensors in specific tasks. Furthermore, perception information from all roles is fused to achieve collective perception.

In collective robots, the perception capability of a robot node *r*_*i*_ is described as a capability vector *C*_*i*_^*r*^ [17]

1 $$C_{i}^{r} = diag\{ \alpha_{i1} ,\alpha_{i2} , \ldots ,\alpha_{im} \} \cdot [c_{1} ,c_{2} , \ldots ,c_{m} ]^{T}$$

where *α*_*ij*_ is the strength of the perception capability *c*_*j*_ for robot *r*_*i*_, and [*c*_1_, *c*_2_,…, *c*_*m*_]^*T*^ is the perception capability set of the collective robots. For a specific task *t*, we can define its relevance vector *C*_*i*_^*t*^ to the perception capability set as

2 $$C_{i}^{t} = diag\{ \beta_{i1} ,\beta_{i2} , \ldots ,\beta_{im} \} \cdot [c_{1} ,c_{2} , \ldots ,c_{m} ]^{T}$$

where *β*_*ij*_ is the relevance of task *t* to the perception capability *c*_*j*_.

micROS adopts a role-based distributed collective perception model for different tasks. When the robots perform collective perception, the role task tree is constructed according to the perception capabilities of the collective robots and the relevance between the capabilities and the given collective perception task. Meanwhile, the role of each robot is assigned. In order to achieve maximal collective efficiency, this role assignment method takes into account the actual perception capabilities of the collective robots at the beginning of producing the role task tree. Thus, it is different from traditional methods, such as those in [18, 19], which directly decompose cooperative tasks to achieve role task trees. The perception task of each node of the role task tree is fulfilled by fusing sensing data of each corresponding robot. Then, perception results of all nodes are further fused for collective perception task. Moreover, in order to achieve globally optimized role assignment strategy and collective perception capability, the quantity and quality of the fulfillment of the collective perception task are evaluated in micROS, based on which the role task tree is dynamically adjusted.

#### Autonomous orientation and collective cognition

micROS is designed to support acquiring, storing and managing collective knowledge. The collective knowledge is further analyzed and utilized to achieve autonomous orientation and collective cognition, which can be used to facilitate the decision of the robots.

Similar to the classification in psychology, collective knowledge is also classified into common sense, experiential knowledge and implicit knowledge by micROS. Collective robots have the common sense including the basic knowledge and rules about inter-robots and external environments that each robots possesses, e.g., communication protocols among collective robots and physical laws, such as Newton’s laws of motion. Experiential knowledge includes the knowledge obtained by practices of individuals of the collective robots and can be accessed by other robots. Implicit knowledge means the knowledge that can be obtained by analyzing all the existing knowledge from the collective robots. How to extract implicit knowledge is one of the research focuses of micROS.

Collective knowledge is stored (i.e., memorized) in common-sense database, experiential-knowledge database and implicit-knowledge database (refer to the cognitive domain in Fig. 3). The three databases are organized into hierarchically structured computer memories [20], in which the collective knowledge is memorized and forgotten following a model similar to human memories [21]. Before a knowledge instance *κ* is accessed by a robot again, the strength *s*_*κ*_ of memorizing *κ* in the robot decreases following a negative exponents curve. When *s*_*κ*_ ≤ *τ*_*f*_, where *τ*_*f*_ is the threshold for forgetting the knowledge on the corresponding hierarchical memory level of *κ*, *κ* will be removed from this memory level to a lower level, which has a larger memory space but longer accessing time. In order to achieve sufficient memory space of knowledge during long-term operation, the knowledge that forgotten by the lowest memory level will be permanently removed from the robot. The hierarchical structured memory is also divided into short-term memory and long-term memory. Those short-term memories, that have been strengthened multiple times, can be promoted as long-term memories.

In order to achieve human–machine collaborated analysis and orientation, different kinds of cognition models are supported in micROS. Techniques, like data mining, will be utilized by micROS to extract collective implicit knowledge. Finally, collective cognition is accomplished by analysis and orientation on top of the three types of collective knowledge.

#### Autonomous decision and collective game

In order to support autonomous decision and planning for the collective robots, micROS supports cooperative and noncooperative game, where robots and even humans are treated as players.

Firstly, micROS supports extraction of the payoff functions and strategy sets of the players. micROS also provides support for building cooperative or noncooperative game models according to whether the players obey the common rules. In a noncooperative game, each player independently makes decisions according to its own objective and intention. In contrast, multiple players may form a coalition in a cooperative game. micROS guarantees information sharing within the coalition, while enforcing the binding contracts [22].

Then, micROS will work out the high-level strategies by solving the cooperative or noncooperative games [24], where various methods can be adopted, e.g., computational intelligence method [24] or simple search method [25]. Afterward, micROS implements specific task and path planning according to the objective of the high-level strategy, while considering the capabilities of each robot in the team. This planning process invokes optimization methods for computing the task allocation and action sequences for individual robots [26, 27].

It should be noticed that humans are also considered to be nodes in the distributed architecture of micROS. And the autonomous decision and collective game module is designed to coordinate human and machine intelligence. So human may participate or intervene the decision process and therefore controls the gaming and decision results.

Consider a scenario where multiple robots cooperate for resource mining. A noncooperative game model can be constructed, where teams of robots may participate as players, and each team aims at maximizing the mining outcome. Human may define rules to affect the strategies made by the collective robots. Firstly, a robot team recovers the environmental information as well as the information of other teams during the collective perception and orientation processes. The information is used for defining the payoff functions and feasible strategies. Then, each team invokes the methods provided by micROS for finding the Nash equilibrium to form the high-level strategy. Finally, each team invokes optimization methods to perform path planning and task allocation for the collective robots.

#### Autonomous action and collective dynamics

Through cooperation and self-organization of multiple robots, coordinated movements may emerge at the collection level. Therefore, collective robots become capable of accomplishing complex tasks, which are impossible for a single robot. According to various action objectives, the environments and the robots’ capabilities, micROS may adopt different collective dynamics models, including Boid model, leader–follower model and graph-based models, while using the corresponding formation-controlling algorithms for coordinated control [28].

When self-organization is required without explicitly defining collective actions, micROS adopts the Boid model, which is characterized by three principles or local actions: collision avoidance, velocity matching and flock centering [29]. Formations of consistent velocity and orientation can be achieved and maintained by local coordination based on these three principles to achieve coordinated control of the collection. In a simpler scenario, where only the velocity matching is considered, the particle model can be used for describing the collective dynamics [30]. Besides, the models based on attract–repulse rules can be used for describing the collision avoidance and flock centering principles, where the potential field method can be used for coordinated control [31].

When the collective action is decided by an individual or some of the robots in the team, the micROS can adopt the leader–follower method for formation control [32]. The leader may be one of the robots in the formation, or a virtual point such as the centroid of the team. Also, multiple leaders may switch roles during the movements.

Graph-based models can be used for quantifying complex network connection relationships in the team, such as sensing, communications and control relationship. Graph theory, especially the algebraic graph theory, can be used for investigating various properties of the robot teams to design the formation and coordinated control mechanisms. In this process, the leader–follower method and the behavior-based methods may be integrated into the framework provided by graph theory.

### Morphable and adaptive mechanism

Collective robot systems live and work in a dynamic and open space, which can be projected into physical, information, cognitive and social domains simultaneously. Moreover, collective robots themselves are extremely heterogeneous and suffer from high hardware and software failure rate. Therefore, some researchers on robotics have reached a consensus that implementing a static, complete and generic robot operating system is a daunting work, if not impossible [33].

**Fig. 5:**
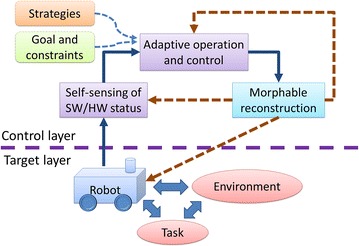
micROS morphable and adaptive framework based on control theory and adaptive software technology

We adopt a morphable and adaptive design in micROS, in order to provide both generality and usability with acceptable system complexity. The resulting system can effectively adapt to heterogeneous collective robots systems and the dynamic and open environments that they operate in. As shown in Fig. 5, the morphable and adaptive mechanism of micROS is based on control theory and adaptive software model [34]. It consists of three modules, i.e., self-sensing of software/hardware status, adaptive operation and control and morphable reconstruction. The three constituent modules together make the robot system in the target layer adaptive to changes of the environment, tasks and the system itself.

#### Self-sensing of software/hardware status

This module is able to detect dynamic, unstructured and unpredictable changes of the robots themselves, tasks and environments. Self-sensing of robot software status is based on program and resource analysis, and the target is the entire ecosystem that the software system of collective robots resides in, including libraries, middleware, services, protocols, models, drivers. Self-sensing of hardware status is achieved by adding extra introspection sensors and corresponding drivers and is able to detect changes in hardware components such as sensors, actuators and processors, as well as unexpected hardware failures.

#### Adaptive operation and control

The adaptive operation and control module takes the detected software and hardware status changes as input. By giving the strategies, goals and constraints, this module may evaluate the system performances after the logical or physical changes occurred in operational environment, resources and hardware devices. Then, decisions on following control operations can be made according to the given strategies, goals and constraints. This module is based on learning-based and experience-based software evolution approaches to achieve intelligent and autonomous software operation and control, including software redeployment and upgrading. Impacts on the robot system are fed back by the previous self-sensing module and used to decide control operations to take in the next step, until the controlled system reaches the target state. These modules form a closed feedback control loop for the adaptive operation and control mechanisms of micROS.

#### Morphable reconstruction

The morphable reconstruction module contains the common functionalities of adaptive software system. It embodies the adaptive control operations triggered by the previous module by performing reconfiguration and reconstruction on the robot software system accordingly. The specific approaches that micROS utilizes include high-level programming languages, formal modeling and analysis tools, automated program transformation, virtual machines supporting cross-platform execution. The operation objects of this module are the entire robot software system, including the self-sensing and adaptive control modules.

## Results and discussion

micROS is an open-source project based on the Robot Operating System (ROS) project [35]. It focuses on morphable resource management and autonomous behavior management, and provides support for collective intelligence. Related codes and resources are available online at http://micros.nudt.edu.cn.

**Fig. 6:**
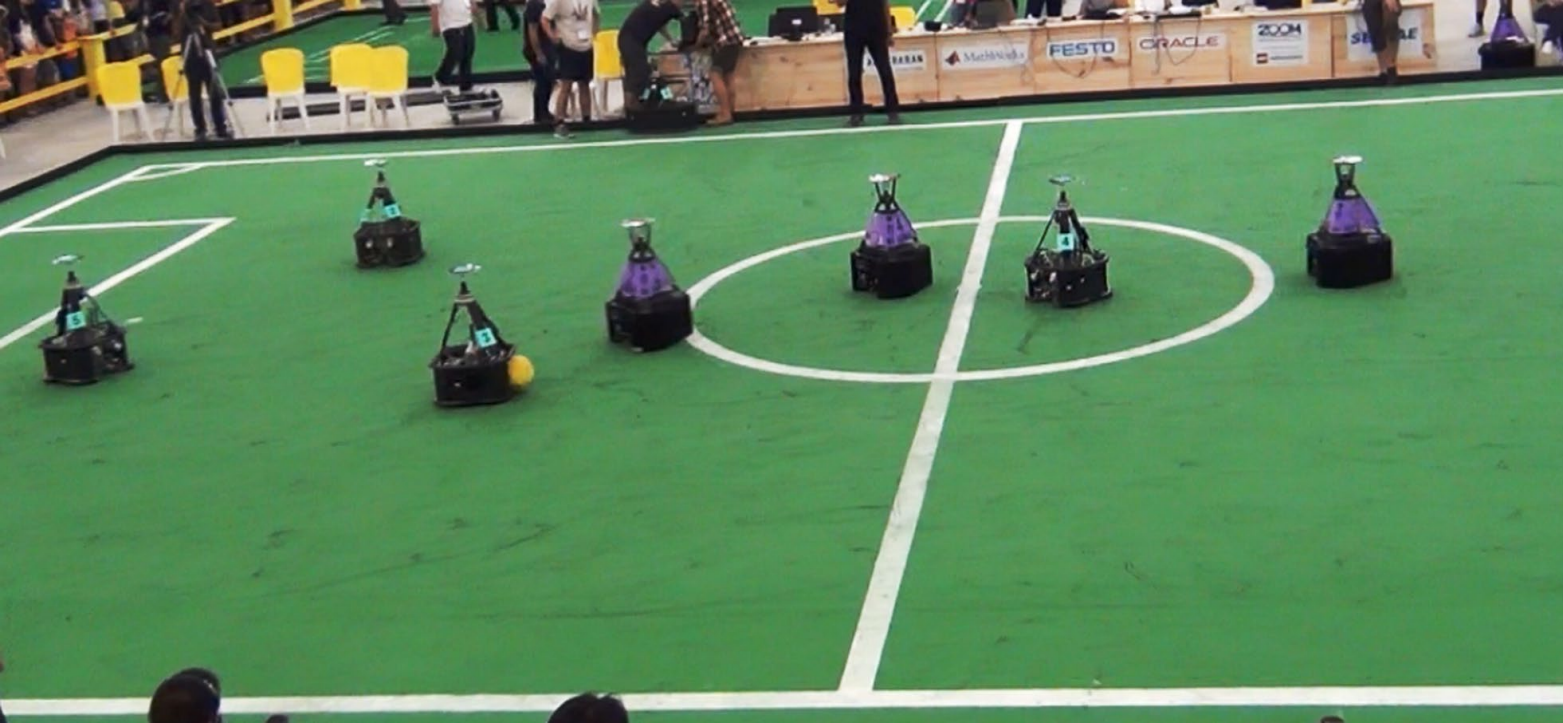
NuBot soccer robots in a cooperative attack

micROS can be applied to various types of robots. We choose a NuBot soccer robot [36] from National University of Defense Technology, which is designed for RoboCup Middle Size League, as an exemplary platform. micROS is installed on every NuBot soccer robot, providing common functionalities such as collective communication, collective perception and collective collaboration. NuBot soccer robots can be found in Fig. 6, with cyan markers.

**Fig. 7:**
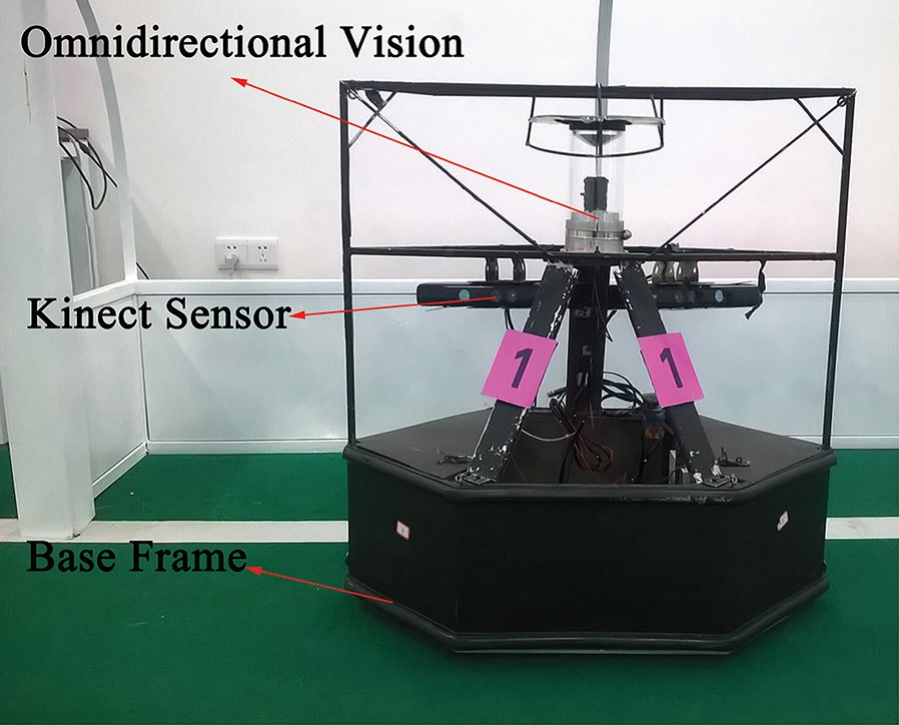
NuBot goalkeeper robot

The NuBot goalkeeper robot, as shown in Fig. 7, is used for experiments on real-time performance of behavior managements of micROS.

Installed on the goalkeeper robot, Kinect sensor is utilized to capture RGB-D images in the frame rate of 30 Hz, which are used to produce color segmentation results and point clouds for localization and motion prediction of the soccer. Thus, the robot can make decisions on whether and how to intercept the soccer. The image processing algorithm is scheduled to process each frame at the rate of 30 Hz, and it must finish before next frame comes. So it is a typical real-time problem.

**Fig. 8:**
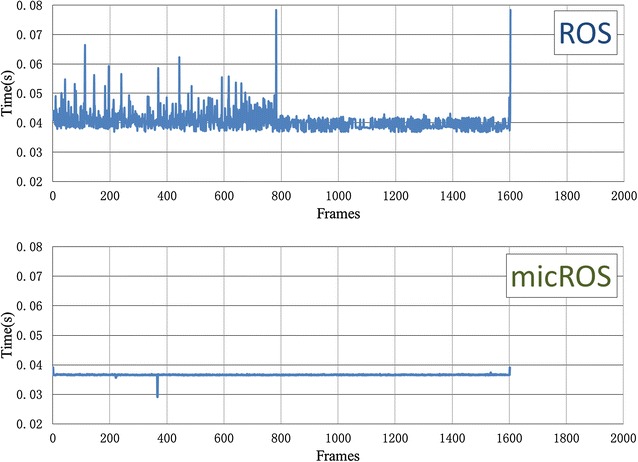
Comparison in image processing time per frame of the Kinect node using ROS (*top*) and micROS (*bottom*)

After deploying micROS on the goalkeeper robot, a significantly improvement in real-time performance, in terms of period jitter, of the Kinect node is achieved compared with the original ROS. Period jitter, which is one of the main benchmarks to evaluate real-time performance, equals to the difference between any actual frame period and the average frame period. The actual frame period is usually not kept stable due to multitasking of the processor. The comparative results are shown in Fig. 8. The maximum period jitters, with the first and last frames excluded to eliminate the startup and cleanup overhead, on ROS and micROS are 0.038018 and 0.000777 s, respectively. We found that the period jitter on micROS is reduced by two orders of magnitude compared with that on ROS. It implies that the image processing task is more accurately started upon each incoming frame. This eliminates the possible delay and leaves as possible as more time for the algorithm. Consequently, the average per frame image processing time of the Kinect node decreases from 0.0403 s on ROS to 0.0366 s on micROS, which implies a 9.2% improvement.

## Conclusions

We present in this paper the design of micROS, a morphable, intelligent and collective robot operating system for collective and cooperative robots, including the architecture, the autonomous behavior and collective intelligence, as well as the morphable and adaptive framework. We also apply micROS on NuBot football robots and achieve two orders of magnitude improvement of real-time performance in terms of period jitter.

We present in this paper a high-level micROS design. Detailed design would be continuously polished in our long-term future work. Based on the open-source ROS project, we will be devoted to implementing a modular and common software platform for usual robot application scenarios. We will also improve micROS by applying it to more kinds of individual robots and collective robots.

## References

[1] Rifkin J (2013). The third industrial revolution: how lateral power is transforming energy, the economy, and the world.

[2] Gates B. A robot in every home. Scientific American (2007). 10.1038/scientificamerican0107-58 17186834

[3] Utz H, Sablatnorg S, Enderle S, Kraetzschmar G (2002). Miro—middleware for mobile robot applications. IEEE Trans Robot Autom.

[4] Makarenko A, Brooks A. Orca: components for robotics. In: Proceedings of IROS’06, Beijing, China; 2006.

[5] Ando N, Suehiro T, Kitagaki K, Kotoku T, Yoon WK. RT-middleware: distributed component middleware for RT (Robot Technology). In: Proceedings of IROS’05, Edmonton, Alberta, Canada; 2005.

[6] Gerkey B, Vaughan R, Howard A. The player/stage project: tools for multi-robot and distributed sensor systems. In: Proceedings of ICAR’03, Coimbra, Portugal; 2003.

[7] Côté C, Brosseau Y, Létourneau D, Raïevsky C, Michaud F (2006). Robotic software integration using MARIE. Int J Adv Rob Syst.

[8] Yoo J, Kim S, Hong S. The robot software communications architecture (RSCA): QoS-aware middleware for networked service robots. In: Proceedings of SICE-ICASE’06; 2006. p. 330–5.

[9] Bruyninckx H, Soetens P, Koninckx B. The real-time motion control core of the Orocos project. In: Proceedings of ICRA’03; 2003. p. 2766–71.

[10] Johns K, Taylor T (2008). Professional microsoft robotics developer studio.

[11] Quigley M, Conley K, Gerkey B, et al. ROS: an open-source robot operating system. In: Proceedings of the workshop on open source software (ICRA’09); 2009.

[12] Aircrew Labor In-cockpit Automation System (ALIAS) Proposers’ Day. 2014. www.fbo.gov/spg/ODA/DARPA/CMO/DARPA-SN-14-32/packages.html.

[13] Robonaut: Home. 2015. http://robonaut.jsc.nasa.gov/.

[14] Horling B, Lesser V (2005). A survey of multi-agent organizational paradigms. Knowl Eng Rev.

[15] Boyd JR. The essence of winning and losing. Retrieved from http://dnipogo.org/john-r-boyd/.

[16] Chaimowicz L, Campos MFM, Kumar V. Dynamic role assignment for cooperative robots. Robotics and automation, 2002. In: Proceedings. ICRA’02. IEEE international conference on. vol. 1. IEEE; 2002.

[17] Lin L, Ji X, Zheng Z (2006). Multi-robot task allocation based on market and capability classification. Robot.

[18] Ge S, Ma D, Huai J (2001). A role-based group awareness model. J Softw.

[19] Zhu Haibin, Zhou MengChu (2006). Role-based collaboration and its kernel mechanisms. IEEE Trans Syst Man Cybern Part C Appl Rev.

[20] Aggarwal A. et al. A model for hierarchical memory. In: Proceedings of the nineteenth annual ACM symposium on theory of computing. ACM; 1987.

[21] Yi F, Ren L (1997). A maths model on studying and recalling. J Math Med.

[22] Serrano R (2005). Fifty years of the Nash program, 1953–2003. Investigaciones Económicas.

[23] Chalkiadakis G, Elkind E, Wooldridge M (2011). Computational aspects of cooperative game theory.

[24] Pavlidis NG, Parsopoulos KE, Vrahatis MN (2005). Computing Nash equilibria through computational intelligence methods. J Comput Appl Math.

[25] Porter R, Nudelman E, Shoham Y (2008). Simple search methods for finding a Nash equilibrium. Games Econ Behav.

[26] Dias MB, Zlot R, Kalra N, Stentz R (2006). Market-based multirobot coordination: a survey and analysis. Proc IEEE.

[27] Jia X, Meng M (2013). A survey and analysis of task allocation algorithms in multi-robot systems. Robot Biomime ROBIO.

[28] Yan Z, Jouandeau N, Cherif AA. A survey and analysis of multi-robot coordination. Int J Adv Robot Syst. 2013;10.

[29] Reynolds CW (1987). Flocks, herds and schools: a distributed behavioral model. ACM SIGGRAPH Comput Graph.

[30] Vicsek T, Czirok A, Ben-Jacob E, Cohen I, Shochet O (1995). Novel type of phase transition in a system of self-driven particles. Phys Rev Lett.

[31] Song P, Kumar V. A potential field based approach to multi-robot manipulation. In: International conference on intelligent robots and systems, (May); 2002. p. 1217–22.

[32] Hu J, Hong Y (2007). Leader-following coordination of multi-agent systems with coupling time delays. Phys A.

[33] Smart W. Is a common middleware for robotics possible? In: Proceedings of the IROS 2007 workshop on measures and procedures for the evaluation of robot architectures and middleware; 2007.

[34] Kokar M, Baclawski K, Eracar YA (1999). Control theory-based foundations of self-controlling software. IEEE Intell Syst.

[35] Quigley M et al. ROS: an open-source robot operating system. In: ICRA workshop on open source software; 2009, vol. 3, no. 3.2.

[36] Xiao J, Lu H, Zeng Z et al. NuBot team description paper 2015. In: Proceedings of RoboCup 2015, CD-ROM; 2015.

